# Supplementary material for: Nanometric Mechanical Behavior of Electrospun Membranes Loaded with Magnetic Nanoparticles
Source: Nanomaterials (Basel). 2023 Apr 1;13(7):1252. doi: 10.3390/nano13071252 (PMC10097362; doi:10.3390/nano13071252)
Supplement: Supplementary file 1 [file nanomaterials-13-01252-s001.zip › nanomaterials-2317593-supplementary.pdf]

nanomaterials

MDPI

Supplementary Materials

Nanometric Mechanical Behavior of Electrospun Membranes Loaded with Magnetic Nanoparticles

Raffaele Longo [1], Luigi Vertuccio [2], Vito Speranza [1], Roberto Pantani [1], Marialuigia Raimondo [1], Elisa Calabrese [1] and Liberata Guadagno [1,*]

1 Department of Industrial Engineering, University of Salerno, Via Giovanni Paolo II 132, 84084 Fisciano, Italy; rlongo@unisa.it (R.L.); vsperanza@unisa.it (V.S.); rpantani@unisa.it (R.P.); mraimondo@unisa.it (M.R.); elicalabrese@unisa.it (E.C.); lguadagno@unisa.it (L. G.)

2 Department of Engineering, University of Campania "Luigi Vanvitelli", Via Roma 29, 81031 Aversa, Italy; luigi.vertuccio@unicampania.it (L. V.)

* Correspondence: lguadagno@unisa.it

XRD Evaluations

The evaluation of the crystallinity by the XRD spectra has been performed by considering the crystalline and the amorphous areas under the diffractometric curve profile, according to Equation S1.

$$X_c = \frac{A_{crystalline}}{A_{crystalline} + A_{amorphous}} \tag{S1}$$

Crystallite sizes were determined using Scherrer Equation (S2):

$$\tau = K * \frac{\lambda}{\beta * \cos(\theta)} \tag{S2}$$

Where τ is the mean size of the crystallite, λ is the wavelength of the X-ray source (0.1542 nm), β is the width of the peak at half maximum intensity and θ is the diffraction angle.

Peak deconvolutions have been done for all of the spectra in order to better handle the data. Based on the Levenberg-Marquardt method [57], a curve resolving algorithm was used for the XRD spectra to separate the individual peaks in the case of unresolved, multi-component bands; the baseline, the band shape, and the number of components were fixed to reduce the number of adjustable parameters and to ensure the uniqueness of the result. The program was then allowed to calculate, by a non-linear curve fitting of data, the height, the full-width half height (FWHH) and the position of the individual components. The applied model is reported in Equation S3. The peak function was a mixed Gauss–Lorentz line shape of the form [58]:

$$f(x) = (1-L) * H * \exp\left(-4 * ln(2) * \left(\frac{x-x_0}{w}\right)^2\right) + L * H * \left(4 * \left(\frac{(x-x_0)}{w}\right)^2 + 1\right)^{-1} \tag{S3}$$

where x_0 = the peak position; H = peak height; w = FWHH; L = fraction of Lorentz character. The results are reported in Figure S1. The fitting model well fits the spectra. The amorphous halo presents the peak around 21° of 2θ, as previously reported in literature. [59] The main parameters of all the curves are reported in Table S1.

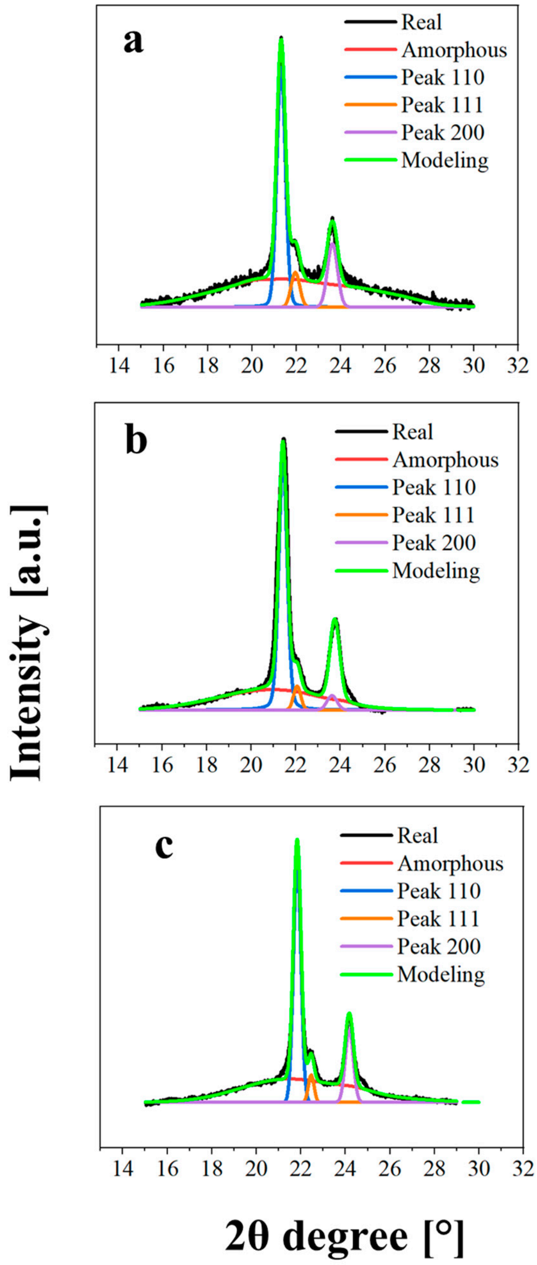

Figure S1. XRD spectra and deconvolutions of the peaks of: a) PCL_0%F-Fe_3O_4; b). PCL_5%F-Fe_3O_4; c) PCL_10%F-Fe_3O_4;

Table S1. Parameters of the fitting for the deconvolutions of the XRD spectra.

**Sample**	R^2
PCL_0%F-Fe_3O_4	0.993
PCL_5%F-Fe_3O_4	0.992
PCL_10%F-Fe_3O_4	0.997

EDX-FESEM Morphology

Energy Dispersive X-ray Analysis (EDX) investigation was performed by a Field Electron Scanning Electron Microscope (FESEM) LEO1525 microscope (Carl Zeiss SMT AG, Oberkochen, Germany) equipped with an EDX detector. EDX maps were obtained after covering the samples with chromium film. In Figure S2a it is reported the morphology of the sample PCL_10%F-Fe_3O_4, whereas in Figure S2b it is reported the elemental map of the iron element. As it is possible to observe, Fe element, that indicates the presence of magnetite, is well distributed along the fiber length in the produced samples.

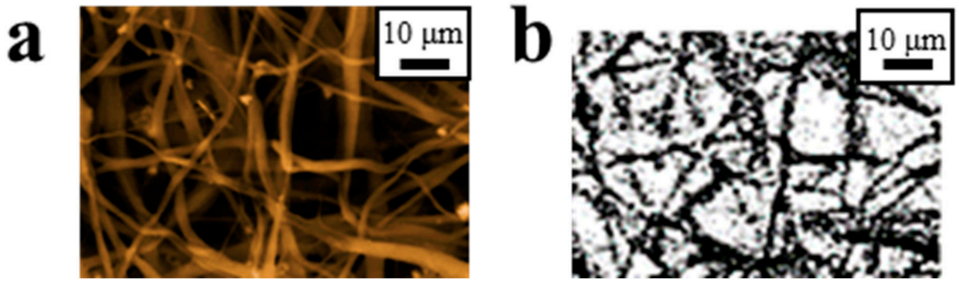

Figure S2. a) EDX-FESEM on PCL_10%F-Fe_3O_4; b) Fe elemental response (black dots).
